# Neuregulin 1 Promotes Glutathione-Dependent Neuronal Cobalamin Metabolism by Stimulating Cysteine Uptake

**DOI:** 10.1155/2016/3849087

**Published:** 2015-12-29

**Authors:** Yiting Zhang, Nathaniel Hodgson, Malav Trivedi, Richard Deth

**Affiliations:** ^1^Department of Pharmaceutical Sciences, Northeastern University, Boston, MA 02115, USA; ^2^Department of Neurology, F.M. Kirby Neurobiology Center, Boston Children's Hospital, Harvard Medical School, 300 Longwood Avenue, Boston, MA 02115, USA; ^3^Department of Pharmaceutical Sciences, Nova Southeastern University, Fort Lauderdale, FL 33328, USA

## Abstract

Neuregulin 1 (NRG-1) is a key neurotrophic factor involved in energy homeostasis and CNS development, and impaired NRG-1 signaling is associated with neurological disorders. Cobalamin (Cbl), also known as vitamin B_12_, is an essential micronutrient which mammals must acquire through diet, and neurologic dysfunction is a primary clinical manifestation of Cbl deficiency. Here we show that NRG-1 stimulates synthesis of the two bioactive Cbl species adenosylcobalamin (AdoCbl) and methylcobalamin (MeCbl) in human neuroblastoma cells by both promoting conversion of inactive to active Cbl species and increasing neuronal Cbl uptake. Formation of active Cbls is glutathione- (GSH-) dependent and the NRG-1-initiated increase is dependent upon its stimulation of cysteine uptake by excitatory amino acid transporter 3 (EAAT3), leading to increased GSH. The stimulatory effect of NRG-1 on cellular Cbl uptake is associated with increased expression of megalin, which is known to facilitate Cbl transport in ileum and kidney. MeCbl is a required cofactor for methionine synthase (MS) and we demonstrate the ability of NRG-1 to increase MS activity, and affect levels of methionine methylation cycle metabolites. Our results identify novel neuroprotective roles of NRG-1 including stimulating antioxidant synthesis and promoting active Cbl formation.

## 1. Introduction

Neuregulin 1 (NRG-1) is an epidermal growth factor- (EGF-) like growth factor that plays critical roles in development of the central nervous system by influencing neuronal differentiation, regulation of neurotransmitter receptor expression, and oligodendrocyte development [1, 2]. Expression of NRG-1 is high in the brain, is lower in peripheral tissues, and gradually decreases with age [3].

Methionine synthase (MS) is a key component of the methionine cycle in one-carbon metabolism and it catalyzes the conversion of homocysteine (HCY) to the essential amino acid methionine, utilizing a methyl group derived from 5-methyltetrahydrofolate. Methionine can further receive an adenosyl moiety from ATP to form S-adenosylmethionine (SAM), which is the universal methyl donor providing methyl groups to more than 250 different methylation reactions including DNA and histone methylation [4] (Figure 6).

MS activity depends upon its cofactor cobalamin (Cbl), also known as vitamin B_12_. Neurologic dysfunction is a primary clinical manifestation of Cbl deficiency and cerebral sequelae of Cbl deficiency include cognitive, memory, and mood disorders [5]. In a recent study we observed more than fivefold lower Cbl levels in postmortem human frontal cortex of schizophrenia subjects when compared to samples from age-matched controls (unpublished results). While the factors leading to this abnormally low Cbl status remain unknown,* in vivo* studies of EGF-knockout neurodegenerative mouse models revealed crosstalk between Cbl metabolism and the EGF system, and EGF can regulate the neurotrophic effects of Cbl in brain [6–8], suggesting the possibility that NRG-1 may play a role in regulating neuronal Cbl metabolism.

There are numbers of naturally occurring Cbl species in the human body, but only methylcobalamin (MeCbl) and adenosylcobalamin (AdoCbl) are biologically active, meaning that only they directly act as enzyme cofactors. Glutathionylcobalamin (GSCbl) is a key intermediate in the biosynthesis of active Cbl species and its formation depends on glutathione (GSH) [9, 10]. GSH is a major antioxidant molecule and a GSH deficit has been suggested to contribute to the etiology of schizophrenia [11]. GSH synthesis in neurons largely relies upon excitatory amino acid transporter 3- (EAAT3-) mediated cysteine uptake [12]. We previously showed that growth factor stimulation of the phosphatidylinositol 3-kinase (PI3K)/Akt signaling pathway promotes EAAT3-mediated cysteine uptake and subsequently increases GSH levels in neurons [13, 14]. Yu et al. recently showed that NRG-1 promotes neuronal EAAT3 expression, particularly in parvalbumin-expressing GABAergic interneurons [15].

Since NRG-1 is capable of initiating the PI3K/Akt signaling cascade [16], we hypothesized that NRG-1 may promote neuronal Cbl metabolism by stimulating EAAT3-mediated cysteine uptake and increasing GSH synthesis, making more GSCbl available for active Cbl species formation. Using the SH-SY5Y human neuroblastoma cell line, we examined the influence of NRG-1 on levels of six individual Cbl species, including the two bioactive Cbls, and the possible involvement of GSH.

## 2. Materials and Methods

### 2.1. Cell Culture

SH-SY5Y human neuroblastoma cells were obtained from ATCC and routinely cultured as proliferative monolayers in 10 cm^2^ tissue culture dishes, with 10 mL of alpha-modified Minimum Essential Medium (*α*-MEM) containing 10% fetal bovine serum (FBS) (HyClone) and 1% penicillin-streptomycin-fungizone (Sigma) at 37°C with 5% CO_2_. For most experiments, cells were plated and incubated in 10% FBS media for 24 h, and then media were replaced with 1% FBS media for another 24 h prior to use.

### 2.2. Cobalamin Quantification

**Figure 1:**
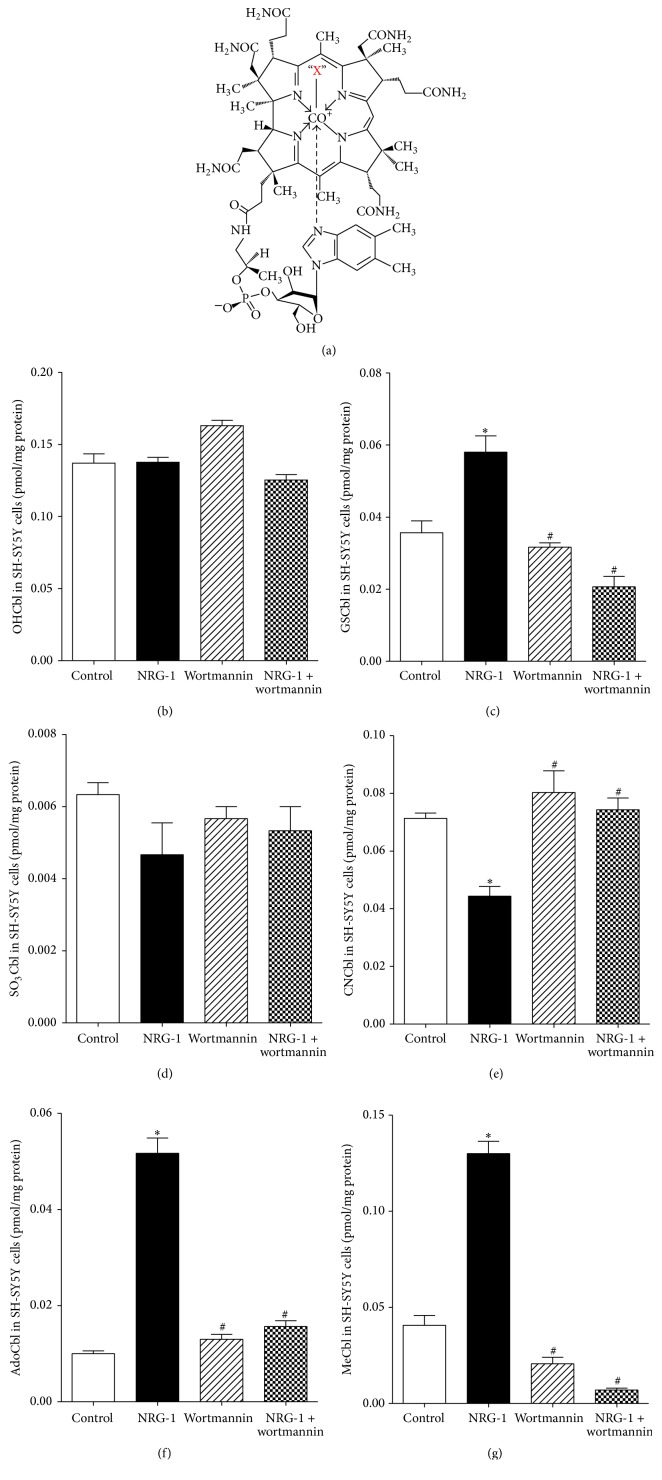
NRG-1 increases biosynthesis of active Cbl species in SH-SY5Y cells. (a) The general structure of Cbl species in which “X” represents various ligands linked to the cobalt atom, giving rise to six different Cbl species measured in SH-SY5Y cells. (b–g) The effects of NRG-1 on six intracellular Cbl species were measured, including OHCbl (b), GSCbl (c), SO_3_Cbl (d), CNCbl (e), AdoCbl (f), and MeCbl (g). Cells were treated for 1 hour with 1 nM NRG-1, 100 nM of the PI3 kinase inhibitor wortmannin, or 1 nM NRG-1 and 100 nM wortmannin combined, *n* = 3. Data represent mean values ± SEM. Asterisks (*∗*) indicate a significant difference (*p* < 0.05) from control group. # indicates a significant difference (*p* < 0.05) from NRG-1 treated group.

Cbl contains a corrin ring which can bind different ligands in the upper *β*-axial position, giving rise to different Cbl species (Figure 1(a)). Cbl extraction and measurement were performed under dim red light due to Cbl's light sensitivity. After pretreatments indicated for individual experiments, culture media were removed and SH-SY5Y cells in the culture dish were washed 3X with 4 mL of Dulbecco's phosphate buffered saline (DPBS). Cells were then lysed with 1.0 mL of 50 mM Tris buffer containing 1% Triton X-100 for 10 min, scraped, and collected in a 1.5 mL microcentrifuge tube. 100 *μ*L of cell lysate was aliquoted for protein quantification by Lowry protein assay [17] and 900 *μ*L of the remaining cell lysate was mixed and incubated with 1.35 mL ice-cold absolute ethanol for 10 min. Protein precipitates were removed by centrifugation at 10,600 RPM for 3 min. The supernatant was dried in a speed-vac, resuspended in 300 *μ*L DPBS, and filtered through a 0.22 *μ*m syringe-driven filter unit. 200 *μ*L of sample was added to a conical micro autosampler vial, blown with nitrogen, capped, and kept at 4°C in the autosampler (ESA model 542). 30 *μ*L of sample was injected into an ESA CoulArray HPLC system equipped with an Agilent Eclipse XD8-C8 (3 × 150 mm; 3.5 *μ*m) reverse-phase C8 column and an Agilent Eclipse XDB-C8 (4.6 × 12.55 mm; 5 *μ*m) guard column. A dual mobile phase gradient elution was used: mobile phase A contains 0.1% acetic acid in water, adjusted to pH 3.5 with 6.0 N ammonium hydroxide, and mobile phase B contains 0.1% acetic acid in acetonitrile. The system was run at a flow rate of 0.6 mL/min at ambient temperature with the following gradients: 0–2 min 0% B, 2–12 min 10% B, 12–15 min 15% B, and 15–35 min 20% B. The system was cleaned from 38 to 44 min with 50% B and was reequilibrated from 44 to 52 min with 0% B. Cbls were measured by electrochemical detection using a boron-doped diamond analytical cell (ESA model 5040) electrochemical detector at an operating potential of 1,000 mV. Peak area analysis was performed using CoulArray software (version 3.06 ESA analysis program) and calculations were based on standard curves generated for each Cbl compound. Sample Cbl levels were normalized against protein content. Cbl extraction and HPLC mobile phase selection were modified based on the protocol developed by Hannibal et al. [18].

### 2.3. Cysteine Uptake

SH-SY5Y cells were plated at 1 million cells per well in six-well tissue culture plates containing 2 mL of 10% FBS supplemented *α*-MEM for 24 h, and then media were replaced with 1% FBS low serum media for another 24 h prior to use. Confluent cells were pretreated with NRG-1 at different concentrations for 1 h. Media were aspirated and cells in each well of the plate were washed with 600 *μ*L of 37°C Hanks buffered salt solution (HBSS). After washing, cells were incubated for 5 min at 37°C with 600 *μ*L of HBSS containing 1 *μ*Ci/1 mL [^35^S]-cysteine, 10 *μ*M unlabeled cysteine and 100 *μ*M dithiothreitol. Radioactive HBSS was removed and cells were washed with ice-cold HBSS for two times. Cells were lysed in 600 *μ*L of dH_2_O, scraped, collected in 1.5 mL microcentrifuge tubes, and sonicated for 10 s. 50 *μ*L of each sample was aliquoted for Lowry protein assay. 200 *μ*L of sample was added to scintillation vials with 4 mL of scintillation fluid, vortexed, and counted for radioactivity. The calculated cysteine uptake was normalized against protein content. This protocol is based on the method developed by Chen and Swanson [19].

### 2.4. Thiol and Thioether Quantification

Thiol and thioether metabolites were measured using HPLC with electrochemical detection. SH-SY5Y cells in 10 cm^2^ tissue culture dishes were pretreated as indicated for individual experiments. After treatment, media were removed and cells were washed 2X with 5 mL of ice-cold HBSS, after which HBSS was aspirated and 600 *μ*L of ice-cold dH_2_O was added. Cells were scraped from the culture dish, suspended in dH_2_O, collected in a 1.5 mL microcentrifuge tube, and sonicated for 15 s on ice. 100 *μ*L of cell lysate was aliquoted for Lowry protein assay. 200 *μ*L of the remaining lysate was mixed with 50 *μ*L 0.4 N perchloric acid, gently blown with nitrogen gas, and centrifuged at 13,000 RPM for 60 min at 4°C. 100 *μ*L of supernatant was added to a conical microautosampler vial, capped, and kept at 4°C in the autosampler cooling tray. 10 *μ*L of each sample was injected into the HPLC system and measured by electrochemical detection. HPLC columns and running conditions were the same as those previously described [13].

### 2.5. qRT-PCR Analysis

SH-SY5Y cells in 10 cm^2^ tissue culture dishes cultured under low serum conditions (1% FBS) were pretreated with 1 nM NRG-1 for 1 h. After treatment, RNA was isolated using the RNAqueous −4PCR kit (Ambion) according to the manufacture's protocol. Extracted RNA was treated with DNase and quantified using a ND-100 NanoDrop spectrophotometer. RNA was reverse transcribed to cDNA with random hexamer primer using the Transcriptor First Strand cDNA Synthesis kit (Roche) according to the manufacture's protocol. Primers were designed using the OligoPerfect Designer (Invitrogen) to have between 50 and 60% GC content, an annealing temperature of 60°C, and a length of 20 bases. Primer sets were checked for primer-dimer formation and each primer was specific for the desired template. The housekeeping gene GAPDH was used as an internal and loading control. Primer sequences were as follows: megalin forward primer, 5′-AGGTCAACAACAACCCTTGC-3′; megalin reverse primer, 5′-TTCTTGCCATCACTTTGCAG-3′; GAPDH forward primer, 5′-GAGTCAACGGATTTGGTCGT-3′; GAPDH reverse primer, 5′-TTGATTTTGGAGGGATCTCG-3′. qRT-PCR was performed on triplicate samples using the LightCycler 480 Real-Time PCR system (Roche). The assay was run in 96-well optical reaction plates and was performed for 45 cycles. Experiment conditions were the same as those previously described [20].

### 2.6. Methionine Synthase Assay

SH-SY5Y cells in six-well tissue culture plates were pretreated as indicated for individual experiments. After treatment, media were removed and cells were washed with 2X with 2 mL of ice-cold HBSS, after which HBSS was aspirated and 500 *μ*L of ice-cold dH_2_O was added into each well. Cells were scraped, suspended in dH_2_O, collected in 1.5 mL microcentrifuge tube, and sonicated for 15 s on ice. 100 *μ*L of cell lysate was aliquoted for Lowry protein assay. The assay was conducted under anaerobic conditions as previously described [21]. In a 5 mL glass vial, 385 *μ*L of the remaining lysate was mixed with 50 *μ*L 1 M potassium phosphate, 25 *μ*L 10 mM HCY, 10 *μ*L 25 mM dithiothreitol, 20 *μ*L 3.8 mM SAM, and 10 *μ*L 5 mM OHCbl. The reaction was initiated by adding 250 *μ*M [^14^C]-5-methyl-THF (2,000 dpm/nmol). Vials were incubated for 1 h at 37°C in a water bath and then placed in boiling water for 2 min to terminate the reaction and then cooled on ice. Reaction mixtures in each vial were passed through a Dowex 1-X8 anion exchange column to separate newly formed [^14^C]-methionine from unreacted [^14^C]-5-methyl-THF. After washing the column with 2 mL dH_2_O, effluents were collected in scintillation vials with 7 mL of scintillation fluid, vortexed, and counted for radioactivity. Nonspecific activity was measured by replacing the 385 *μ*L of cell lysate with dH_2_O under identical experimental conditions. MS activity was calculated by subtracting nonspecific activity and being normalized to protein content [14].

### 2.7. Statistical Method

Statistical analyses were carried out using Graph Pad Prism version 5.01. Results were expressed as mean ± SEM. Two-tailed Student's *t*-test and one-way analysis of variance (ANOVA) with Tukey's* post hoc* test were used to evaluate statistical significance.

## 3. Results

### 3.1. NRG-1 Increases Biosynthesis of Active Cbl Species in SH-SY5Y Cells

Among different Cbl species, only MeCbl and AdoCbl are metabolically active, functioning as cofactors for the cytoplasmic enzyme MS and the mitochondrial enzyme methylmalonyl-CoA mutase, respectively. Other Cbl species need to be converted to these metabolically active species before being utilized by enzymes. To investigate the effects of NRG-1 on neuronal Cbl mechanism, especially the effects on synthesis of bioactive Cbl species, we utilized a novel HPLC/electrochemical detection-based assay which is able to sensitively and accurately quantify six naturally occurring Cbl species in human cultured neuronal SH-SY5Y cells, including hydroxocobalamin (OHCbl), GSCbl, sulfitocobalamin (SO_3_Cbl), cyanocobalamin (CNCbl), AdoCbl, and MeCbl.

Treating SH-SY5Y cells with 1 nM NRG-1 for one hour resulted in significantly increased levels of metabolically active AdoCbl and MeCbl, while decreasing the level of CNCbl without changing the level of SO_3_Cbl (Figure 1). CNCbl is not biologically active but is taken up by cells and converted to active Cbls. Thus the deceased CNCbl level along with increased AdoCbl and MeCbl indicated that NRG-1 has the ability to promote conversion of inactive Cbl derivatives to active ones. Interestingly, NRG-1 also significantly increased the level of GSCbl which is considered to be a key intermediate in the biosynthesis of AdoCbl and MeCbl [9] (Figure 1(c)), suggesting that NRG-1 is likely to promote synthesis of active Cbl species by stimulating formation of their precursor GSCbl. Formation of GSCbl depends upon the reaction of OHCbl with GSH [9], suggesting that NRG-1 may achieve these effects by enhancing GSH concentration. Thus, we hypothesized that NRG-1 promoted the formation of active Cbl species via an influence on GSH status.

### 3.2. Biosynthesis of Active Cbl Species Is GSH-Dependent

**Figure 2:**
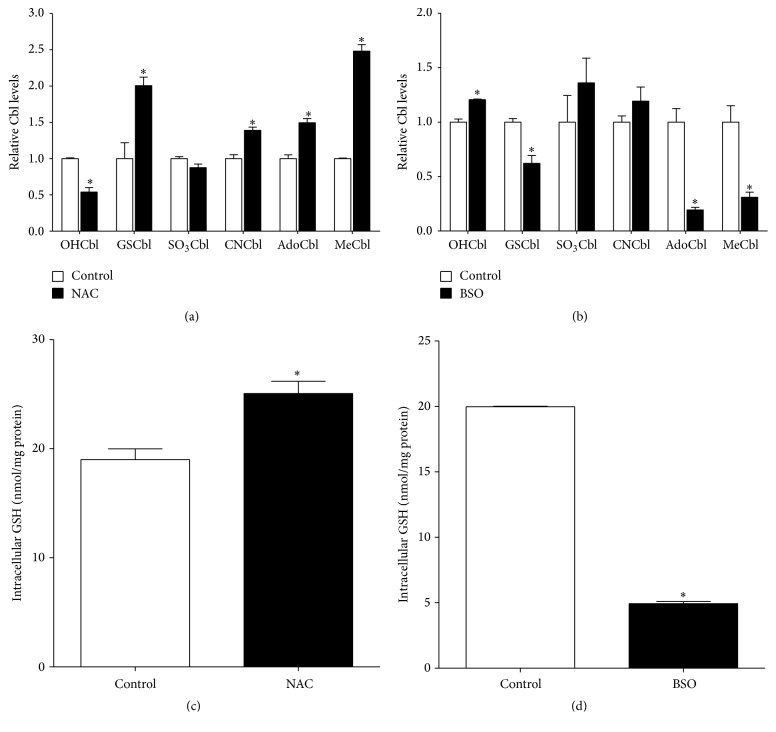
Biosynthesis of active Cbl species is GSH-dependent. (a) 24 hr treatment of NAC (100 mM), an acetylated derivative of cysteine, increased levels of active Cbl species AdoCbl and MeCbl and levels of CNCbl and GSCbl, while the level of OHCbl was decreased, *n* = 4. (b) Four hr treatment with the GSH synthesis inhibitor BSO (1 mM) decreased levels of GSCbl, AdoCbl, and MeCbl, while it increased the level of OHCbl, *n* = 4. All data represent the mean ± SEM and Cbl levels were normalized against control values. (c) 24 hr treatment of NAC (100 mM) increased the GSH level, *n* = 3. (d) Four hr treatment of BSO (1 mM) decreased the GSH level, *n* = 3. Asterisks (*∗*) indicate a significant difference (*p* < 0.05) from control group.

To test our hypothesis that NRG-1 promoted the formation of AdoCbl and MeCbl by increasing the GSH level, we first determined whether synthesis of active Cbl species was GSH-dependent. Therefore, intracellular GSH levels were manipulated to test if changes in GSH influenced Cbl status. First, we treated SH-SY5Y cells with N-acetylcysteine (NAC), a derivative of cysteine that is readily taken up by cells and then rapidly deacetylated, making cysteine available for GSH synthesis [22, 23]. NAC treatment increased the level of GSCbl and decreased the level of OHCbl (Figure 2(a)), indicating that newly synthesized GSH from NAC reacts with OHCbl to form GSCbl. Additionally, increase in AdoCbl and MeCbl in response to NAC addition is consistent with augmented GSH-dependent synthesis of active Cbl species. On the other hand, cells were also treated with buthionine sulfoximine (BSO), an irreversible inhibitor of *γ*-glutamylcysteine synthetase to block GSH synthesis from cysteine [24]. Accordingly, we found that a 4-hour BSO treatment affected Cbl levels in a manner opposite to NAC (Figure 2(b)), consistent with GSH-dependent synthesis of active Cbl species. Overall, these results support the conclusion that biosynthesis of active Cbl species depends on GSH status.

### 3.3. NRG-1 Promotes GSH Synthesis by Stimulating EAAT3-Mediated Cysteine Uptake

**Figure 3:**
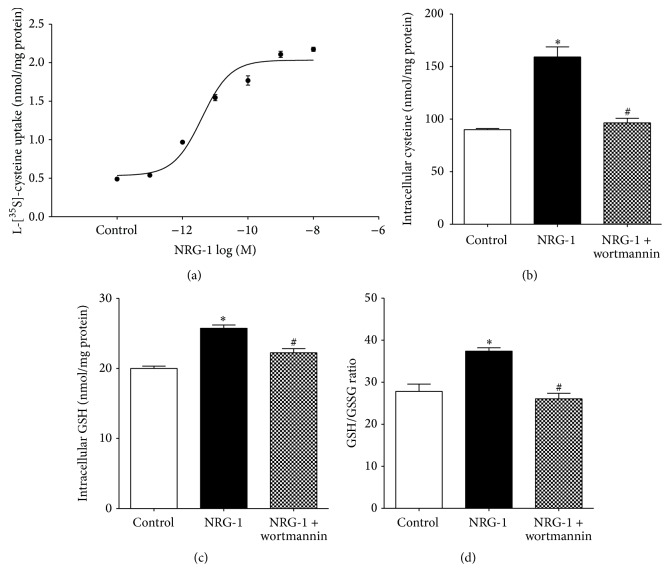
NRG-1 promotes GSH synthesis by stimulating EAAT3-mediated cysteine uptake. (a) EAAT3-mediated [^35^S]-cysteine uptake was increased by NRG-1 with an EC_50_ of 3.91 pM. Cells were treated with 1 nM NRG-1 for 1 hr, *n* = 3. Intracellular levels of cysteine (b) and GSH (c) and the ratio of GSH/GSSG (d) were increased by 1 hr NRG-1 (1 nM) treatment. These effects were blocked by pretreatment with the PI3 kinase inhibitor wortmannin (100 nM), *n* = 3. Data represent mean values ± SEM. Asterisks (*∗*) indicate a significant difference (*p* < 0.05) from control group. # indicates a significant difference (*p* < 0.05) from NRG-1 treated group.

Next we examined whether NRG-1 was able to stimulate GSH synthesis. GSH is a tripeptide composed of cysteine, glutamate, and glycine, synthesized in most mammalian cells, and cysteine is the rate-limiting precursor for its synthesis. Intracellular cysteine is provided either through the intracellular transsulfuration pathway or by uptake of extracellular cysteine. In the transsulfuration pathway HCY is converted to cysteine via a cystathionine intermediate; however, this pathway is limited in human neurons due to low activity of cystathionine-*γ*-lyase [25]. Thus in neurons roughly 90% of cysteine uptake is provided by the EAAT3 transporter [26]. We previously showed that EAAT3 is the primary cysteine transporter in SH-SY5Y cells and EAAT3-mediated cysteine uptake is increased via PI3K activation [13]. To investigate the dose-dependent effect of NRG-1 on cysteine uptake, SH-SY5Y cells were treated with NRG-1 at different concentrations (Figure 3(a)). NRG-1 significantly stimulated [^35^S]-radiolabeled cysteine uptake 4-fold with an EC_50_ of 3.91 pM and the maximal stimulatory effect was achieved at the concentration of 1 nM. This observation was further supported by HPLC results showing that 1 nM NRG-1 increased the intracellular cysteine level, GSH level, and the ratio of reduced GSH to oxidized glutathione (GSSG) (Figures 3(b)–3(d)). The GSH/GSSG ratio is an important indicator of cellular redox status and its augmentation suggests that NRG-1 shifts the cellular environment to be more reducing. In order to investigate involvement of PI3K/Akt signaling, cells were treated with the PI3 kinase inhibitor wortmannin. Wortmannin eliminated the effects of NRG-1, confirming that NRG-1 stimulates GSH synthesis by activating the PI3K signaling pathway (Figures 3(b)–3(d)). Moreover, consistent with our observation that NRG-1 promoted Cbl metabolism by increasing GSH synthesis, NRG-1-induced changes in Cbl status were also blocked by pretreatment with wortmannin (Figure 1).

### 3.4. NRG-1 Increases mRNA Level of Megalin

**Figure 4:**
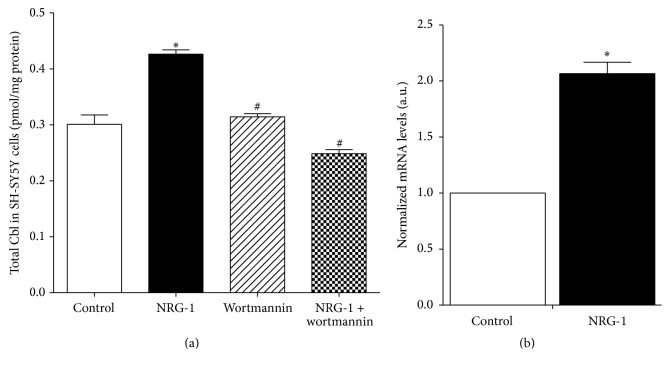
NRG-1 increases mRNA level of megalin. (a) NRG-1 (1 nM) increased total Cbl level in SH-SY5Y cells and this stimulatory effect was blocked by pretreatment with the PI3 kinase inhibitor wortmannin (100 nM), *n* = 3. Total Cbl was calculated by summing individual Cbl values presented in Figure 1. (b) NRG-1 (1 nM) increased the mRNA level of megalin. *n* = 3. Data represent mean values ± SEM. Asterisks (*∗*) indicate a significant difference (*p* < 0.05) from control group. # indicates a significant difference (*p* < 0.05) from NRG-1 treated group.

NRG-1 treatment (1 nM; 1 hr) caused a significant increase in total cellular Cbl level, calculated as the six Cbl species combined (Figure 4(a)). Since human cells are incapable of synthesizing Cbl and must completely reply on extracellular Cbl sources, we hypothesized that the total Cbl level augmentation caused by NRG-1 resulted from increased cellular Cbl uptake. Megalin is an endocytic receptor with a high affinity for the Cbl transport protein transcobalamin II that can mediate Cbl cellular uptake through endocytosis of the transcobalamin II-Cbl complex [27]. Megalin is expressed by neurons [28] and megalin-knockout mice display severe abnormalities in brain development [29]. Thus we evaluated the mRNA level of megalin by qRT-PCR and found that megalin was expressed by SH-SY5Y cells and its mRNA level was significantly increased by a 1-hour treatment with 1 nM NRG-1 (Figure 4(b)). This action of NRG-1 was blocked by pretreatment with wortmannin, suggesting that NRG-1 may stimulate cellular Cbl uptake by increasing megalin expression via the PI3K/Akt signaling pathway (Figure 4(b)).

### 3.5. NRG-1 Stimulates MS Activity and Increases Methylation Capacity

**Figure 5:**
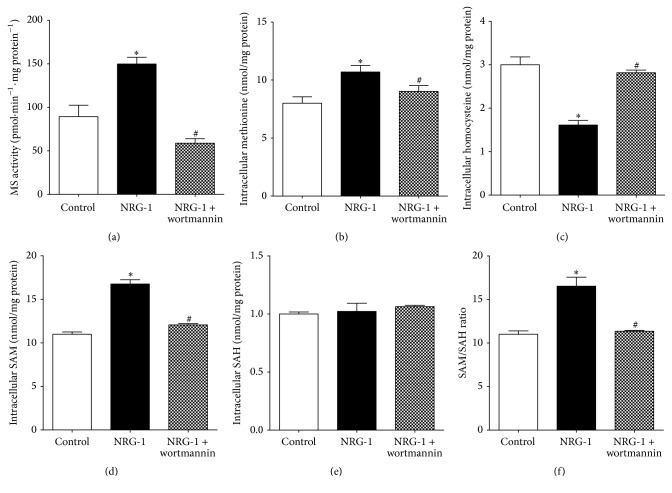
NRG-1 stimulates MS activity and increases methylation capacity. (a) NRG-1 (1 nM) stimulated MS activity and this effect was blocked by wortmannin (100 nM), *n* = 3. (b–f) One hr NRG-1 (1 nM) treatment increased methionine level (b) and decreased HCY level (c); NRG-1 (1 nM) increased cellular methylation capacity by increasing SAM level (d) without changing SAH status (e), leading the increased SAM/SAH ratio (f), *n* = 3. Data represent mean values ± SEM. Asterisks (*∗*) indicate a significant difference (*p* < 0.05) from control group. # indicates a significant difference (*p* < 0.05) from NRG-1 treated group.

MeCbl is the cofactor for MS and MS activity is MeCbl-dependent [30, 31]. Since NRG-1 increased the level of MeCbl (Figure 1(g)), we investigated whether NRG-1 affects MS activity. NRG-1 increased MS activity approximately twofold and this effect was blocked by wortmannin (Figure 5(a)), consistent with our previous finding that MS activity is stimulated by PI3K signaling activation [14]. MS catalyzes the conversion of HCY to methionine which, in the presence of ATP, can be further converted to SAM. SAM is a universal methyl donor and it is converted to S-adenosylhomocysteine (SAH) after donating a methyl group. The ratio of SAM/SAH is an essential determinant of cellular methylation capacity [32]. NRG-1 treatment increased methionine and decreased HCY, confirming the stimulatory effect of NRG-1 on MS activity (Figures 5(b) and 5(c)), while the increased level of SAM level and SAM/SAH suggests a role for NRG-1 in raising cellular methylation capacity (Figures 5(d)–5(f)). Together these results show that NRG-1 stimulates MeCbl-dependent MS activity with consequences for methylation-dependent reactions.

## 4. Discussion

**Figure 6:**
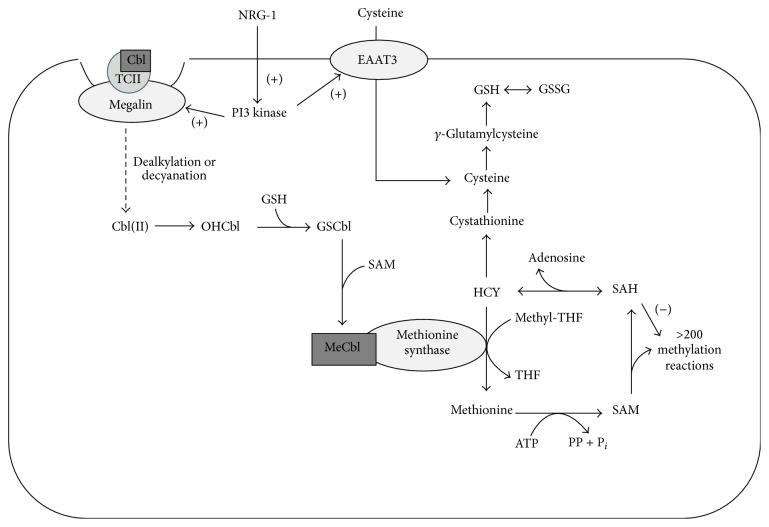
Proposed pathways by which NRG-1 stimulates GSH synthesis and Cbl metabolism. NRG-1 promotes megalin-involved Cbl uptake after which Cbl undergoes dealkylation or decyanation, giving rise to Cbl(II) and subsequently to OHCbl. NRG-1 also stimulates EAAT3-mediated cysteine uptake and promotes GSH synthesis by activating the PI3K signaling pathway. GSH reacts with OHCbl to form GSCbl, which then receives a methyl group from the methyl donor SAM to produce MeCbl. MeCbl functions as the cofactor for MS and increased MS activity promotes the methionine cycle of methylation. The dotted line indicates that multiple steps are involved.

NRG-1 is a neurotrophic factor expressed in both the developing and adult brain. It plays essential roles in many aspects of neural development, including supporting neuron migration, guiding oligodendrocyte development, stimulating synapse formation, and regulating peripheral nerve myelination [2, 33]. Here, we report previously unidentified actions of NRG-1 to increase cysteine uptake, enhance antioxidant synthesis, and promote Cbl metabolism in human cultured neuronal cells (Figure 6).

Oxidative stress is a common pathological hallmark of many neurological disorders and it induces oxidative damage and neurodegeneration under a variety of circumstances [34]. NRG-1 has been shown to protect neurons against oxidative stress initiated by different agents, such as hydrogen peroxide, organophosphates, and MPP+ [35–37], and it has been recognized that NRG-1 exerts this neuroprotective effect through activation of the canonical PI3K/Akt signaling pathway [35, 37].

Our current results provide additional mechanistic insights as to how NRG-1 activation of the PI3K/Akt pathway provides neuroprotection against oxidative stress by demonstrating that NRG-1 stimulates cysteine uptake, leading to increased synthesis of GSH, the primary antioxidant in neuronal cells (Figure 3). Cysteine is the rate-limiting precursor for GSH synthesis illustrated by the fact that the intracellular cysteine concentration approximates the apparent *K*_*m*_ value of *γ*-glutamylcysteine synthetase [38]. EAAT3-null mice display neuronal GSH deficiency and develop behavioral abnormalities [12], indicating that EAAT3-mediated cysteine uptake is essential for maintaining neuronal GSH homeostasis. Under basal conditions, only about 20% of EAAT3 protein is present at the cell surface membrane, with the majority of transporters being sequestered in cytoplasmic vesicles [39]. Stimulation of the PI3/Akt kinase signaling pathway can translocate EAAT3 from cytoplasm to the cellular surface and increase cysteine uptake, similar to the ability of insulin to translocate glucose transporter to the cell surface [40]. Many growth factors are able to initiate PI3K/Akt signaling, such as insulin-like growth factor 1 (IGF-1), which stimulates EAAT3-mediated cysteine uptake with an EC_50_ of 0.98 nM [13], while in this study NRG-1 exhibited EC_50_ of 3.91 pM (Figure 3(a)), almost 400-fold more potent than IGF-1. However, Yu et al. reported that NRG-1 increases EAAT3 expression and glutamate uptake in C6 glioma cells and rat-derived primary cortical neurons with EC_50_ of 1 nM [15]. Cell-type, species differences may contribute to the higher potency we observed in human neuronal cells, but this requires further investigation. Overall it is apparent that NRG-1 may play a powerful role in augmenting antioxidant status in neurons expressing its ErbB4 receptor.

Oxidative stress impairs MS activity and MS activity is highly sensitive to cellular oxidative status because of its Cbl cofactor. The cobalt atom in Cbl structure can exist in different oxidation states. In its cob(I)alamin (Cbl(I)) state, it contains a pair of electrons in the *dz* ^2^ orbital oriented perpendicularly to the plane of the corrin ring, which makes it an excellent nucleophile [25]. In the event of oxidative stress, Cbl(I) is easily oxidized by loss of a single electron, leading to the formation of Cbl(II). Cbl(II) is unable to actively function as the cofactor for MS, temporarily halting methylation of HCY and promoting its diversion toward GSH synthesis to combat the accumulation of reactive oxygen species [32]. In this manner Cbl functions as a sensor, helping cells maintain redox status within a homeostatic range. The brain exists in a closed compartment surrounded by the blood-brain barrier, creating an opportunity for a unique redox environment. Brain consumes approximately 20% of the basal oxygen consumption but contains only limited amounts of GSH, making it more susceptible to oxidative insult than the rest of the body [41]. In addition, alternative mRNA splicing of MS is particularly active in human brain, which allows formation of multiple MS protein variants from a single gene [42]. The cap domain is one among five domains of MS and it normally shields the upper face of Cbl to partially protect it from oxidation. One common alternatively spliced form of MS identified in human brain lacks the cap domain, which may increase the vulnerability of Cbl(I) to oxidation and enhance the sensitivity of MS to reactive oxidative species [42]. These features combine to make the brain more vulnerable to oxidative challenges and consequently increase the redox regulatory influence of NRG-1.

Cbl has the most complex structure of all known organic cofactors and its core structure includes a corrin ring in which the central cobalt atom is equatorially tethered to four nitrogen atoms. The upper axial position of cobalt can be occupied by several different ligands, giving rise to different Cbl species (Figure 1(a)). In this study we have measured the effects of NRG-1 on six major naturally occurring Cbl species in SH-SY5Y cells (Figure 1). OHCbl is a hydroxylated form of Cbl and is often used for cyanide toxicity and Cbl deficiency treatment [5, 43]. In cytoplasm Cbl(II) resulting from decyanation or dealkylation of dietary Cbl is converted to OHCbl under aerobic conditions, which further reacts with GSH to produce active Cbl species [44, 45]. Increased GSCbl shows that NRG-1 stimulated the reaction between OHCbl and GSH (Figures 1(b) and 1(c)). CNCbl is widely used in pharmaceuticals and supplements due to its high stability [5, 46]. Because it is also typically used as the source of vitamin B_12_ in cell culture media including *α*-MEM we also used it for SH-SY5Y cell culture [47, 48]. The decrease in CNCbl caused by NRG-1, along with increased AdoCbl and MeCbl, suggests that NRG-1 promotes the conversion of inactive CNCbl to active AdoCbl and MeCbl (Figures 1(e)–1(g)). Results also show that a 1-hour NRG-1 treatment did not influence the level of SO_3_Cbl (Figure 1(d)). SO_3_Cbl is a Cbl source from food, but its specific biological function has yet to be identified [49]. Taken together, these findings suggest that NRG-1 promotes the synthesis of active Cbl species from inactive ones.

One potential mechanism of NRG-1 stimulated active Cbl synthesis is to raise the antioxidant GSH concentration. Like other studies, we found that formation of active Cbl species was GSH-dependent (Figure 2) [10, 45, 50]. Food-derived Cbl species undergo dealkylation or decyanation where their original cobalt-attached *β* ligands are replaced with methyl or adenosyl groups to form active Cbl species, and dealkylation of alkylcobalamins requires the thiolate of GSH for nucleophilic displacement [45]. Although decyanation of CNCbl normally utilizes NADPH instead of GSH as an electron donor, a recent study suggests that GSH also plays a role in decyanation by increasing CNCbl binding affinity and shifting the equilibrium to a decyanation-favored active state [50]. Besides reacting with OHCbl to form the precursor GSCbl, these data suggest that GSH also play a role in promoting both dealkylation and decyanation of dietary Cbl species.

Our studies show that NRG-1 increases the Cbl uptake. Transport and metabolism of Cbl are tightly regulated in the human body. Once it enters the GI tract, diet-derived Cbl is bound and protected by a series of chaperone and transport proteins including megalin [51]. Megalin mediates endocytosis of carrier protein-bound Cbl and it is also present in placenta and the choroid plexus, suggesting its key roles in both fetal Cbl supply and brain Cbl uptake [52]. Indeed, megalin-deficient embryonic mice display abnormal formation of the forebrain and its derived structures, giving rise to a holoprosencephaly phenotype [29]. Prior studies have shown that activation of PI3K/Akt increases both mRNA and protein levels of megalin [53]. As an endocytic receptor for multiple ligands, megalin also actively participates in amyloid-*β* (A*β*) clearance [54], and a recent study showed that blood-brain barrier megalin-knockout mice model developed neurodegeneration and Alzheimer's disease-like symptoms [55]. Therefore, in addition to increasing Cbl uptake, NRG-1 might be also involved in other megalin-mediated endocytosis events, such as A*β* clearance.

Schizophrenia is a complex mental disorder, with pathological features such as abnormal DNA methylation, altered glutamate synaptic transmission, and increased oxidative stress. Decreased levels of NRG-1 and its receptor ErbB4 are observed in prefrontal cortex of patients with schizophrenia and the NRG-1 gene has been identified as a leading susceptibility locus for schizophrenia [56, 57]. However, the specific mechanism of how impaired NRG-1 signaling is involved in the etiology of schizophrenia has not been fully elucidated. Low Cbl levels have been found in patients suffering from schizophrenia and other neuropsychiatric disorders [58–60] and Cbl supplementation has shown some therapeutic benefit to improve neurological symptoms [61, 62]. Additionally, evidence suggests that PI3K signaling is impaired in the brains of patients with schizophrenia and its impairment is closely related to schizophrenia-associated genetic variants in ErbB4, the NRG-1 receptor [63]. The role of NRG-1 in antioxidant and Cbl metabolism identified in this study may contribute to the etiology of schizophrenia and recognition of this mechanism may lead to novel treatment approaches.

In summary, our study demonstrates that NRG-1 potently activates EAAT3-mediated cysteine uptake and GSH formation and promotes redox-dependent synthesis of bioactive Cbl species in human neuronal cells via the PI3K/Akt signaling pathway, which in turn stimulates MS activity, allowing redox control over methylation status. In addition, we show that NRG-1 increases megalin mRNA, which may contribute to increased neuronal Cbl uptake and other megalin-mediated actions. Together these findings identify novel neuroprotective effects of NRG-1, providing a potential mechanistic link between impaired NRG-1 signaling and neurological disorders such as schizophrenia.

## Abbreviations

A*β*: Amyloid-*β*
AdoCbl: Adenosylcobalamin
BSO: Buthionine sulfoximine
Cbl: Cobalamin
CNCbl: Cyanocobalamin
EAAT3: Excitatory amino acid transporter 3
EGF: Epidermal growth factor
GSCbl: Glutathionylcobalamin
GSH: The reduced glutathione
GSSG: The oxidized glutathione
HCY: Homocysteine
IGF-1: Insulin-like growth factor 1
MeCbl: Methylcobalamin
MS: Methionine synthase
NAC: N-Acetylcysteine
NRG-1: Neuregulin 1
OHCbl: Hydroxocobalamin
PI3K: Phosphatidylinositol 3-kinase
PLM: Phospholipid methylation
PV+: Parvalbumin-expressing
SAM: S-Adenosylmethionine
SAH: S-Adenosylhomocysteine
SO_3_Cbl: Sulfitocobalamin
THF: Tetrahydrofolate.
